# Determinants of poor sleep quality in hemodialysis patients: a multicenter cross sectional study in Sri Lanka

**DOI:** 10.3389/fmed.2026.1780694

**Published:** 2026-03-18

**Authors:** Dishani Weerakoon, Ranga Perera, Ruvini Ambillapitiya, Sanara Waidyasuriya, Medhake Herath, Ranga Tudugala, Ravi Bodhipaksha, Chintha Gunaratne, Nadeeka Perera, Nalaka Herath, Damayanthi Dassanayake, Steven Albert, Kithsiri Jayasekara

**Affiliations:** 1 Faculty of Allied Health Sciences, General Sir John Kotelawala Defence University, Werahera, Sri Lanka; 2 Faculty of Allied Health Sciences, University of Peradeniya, Peradeniya, Sri Lanka; 3 Royal Sussex Country Hospital, Brighton, United Kingdom; 4 Medical Laboratory, National Nephrology Specialized Hospital, Polonnaruwa, Sri Lanka; 5 National Renal Disease Prevention and Research Unit, Ministry of Health, Colombo, Sri Lanka; 6 Nephrology Unit, Colombo North Teaching Hospital, Ragama, Sri Lanka; 7 Department of Behavioral and Community Health Sciences, Graduate School of Public Health, University of Pittsburgh, Pittsburgh, PA, United States

**Keywords:** dialysis recovery time, dialysis vascular access, hemodialysis, Pittsburgh Sleep Quality Index, sleep quality

## Abstract

**Background:**

Sleep quality is a common and often overlooked clinical issue among hemodialysis patients, which can affect physical and psychological quality of Life. This study intended to evaluate sleep quality and its determinants among patients receiving hemodialysis.

**Methods:**

A cross-sectional study was conducted at three hemodialysis centers in Anuradhapura and Polonnaruwa districts of Sri Lanka, including a random sample of 310 maintenance hemodialysis patients. The Pittsburgh Sleep Quality Index (PSQI) was used to assess the sleep quality of the patients. Socio-demographic information, dialysis and medical data, dietary and lifestyle information were also recorded. Several laboratory investigations were conducted before and after dialysis. Dialysis adequacy was calculated using single pool Kt/V. Multiple linear regression was performed to identify significant predictors of poor sleep quality.

**Results:**

A significant majority (83.3%) of the population exhibited poor sleep quality. According to the PSQI, poor sleep latency and low sleep duration were key contributing factors. A significant association was observed between the sleep quality of patients and the number of dialysis sessions per week (*p* = 0.001), serum phosphorus level (*p* = 0.016) and several dialysis side effects. Total number of dialysis side effects and complications (*p* < 0.01), recovery time (*p* < 0.01) and post dialysis urea concentration (*p* = 0.027) depicted a significant positive correlation, while dialysis adequacy (*p* = 0.001), post dialysis sodium level (*p* = 0.006) and hemoglobin level (*p* = 0.005) had a notable negative correlation with global PSQI score of patients. A significant difference in overall sleep quality was observed among the categories of patients’ dialysis vintage (*p* = 0.032), appetite (*p* < 0.01) and dialysis center (*p* < 0.01). Dialysis vascular access depicted a marginal significance.

**Conclusion:**

The study revealed a striking prevalence of poor sleep quality in the population. According to the multiple linear regression model, dialysis center, dialysis access, number of dialysis side effects and complications, appetite and post dialysis serum urea level were identified as significant predictors of poor sleep quality in the population. Subsequent research should develop appropriate interventions to address inadequate sleep quality among hemodialysis patients.

## 1 Introduction

Chronic kidney disease (CKD) is acknowledged as a critical and growing public health concern all over the world. Due to global population expansion and ageing, the prevalence and incidence of CKD have increased by 40% over the last 30 years (1). Contrary to cardiovascular disease, stroke, and respiratory illness, the mortality rate associated with CKD has been increasing (2). According to current statistics, kidney disease ranks as the third most rapidly increasing cause of death worldwide. It is the sole non-communicable disease with a persistent increase in age-adjusted mortality. By 2040, CKD is anticipated to rank as the fifth leading cause of Years of Life Lost (YLL) worldwide (3). Most importantly, research has revealed an increasing trend in the prevalence and mortality of CKD over the next decade, which poses a major risk to future global health (1).

Additionally, it is estimated that approximately 850 million people worldwide suffer from kidney disease, primarily in low- and lower-middle-income countries, with many lacking access to diagnosis, prevention, or treatment for the condition (2). As a lower middle-income country, Sri Lanka has been burdened by chronic kidney disease (CKD) caused by diabetes and hypertension; particularly over the past two decades, chronic kidney disease of uncertain aetiology (CKDu) has challenged both the healthcare system and the country’s economic stability. In 2020, 164,000 people were diagnosed with chronic kidney disease, with 10,500 consequent deaths linked to kidney failure and kidney diseases, which tragically resulted in a vast increase in the annual death rate (4).

When CKD progresses to End Stage Kidney Disease (ESKD), patients need a Renal Replacement Therapy (RRT) such as Hemodialysis (HD), Chronic Ambulatory Peritoneal Dialysis (CAPD) or a kidney transplant to ensure survival. There are nearly 4 million people worldwide who are now surviving on RRT, where hemodialysis is still the most frequent kind of kidney replacement therapy, accounting for roughly 69% of all RRT, and 89% of all dialysis (5).

Nevertheless, patients undergoing maintenance haemodialysis endure an intense burden, as the side effects related to haemodialysis treatment impact their physical and emotional health, ultimately diminishing their overall quality of life (6). Among several factors, there is a growing body of evidence that declares the sleep quality of haemodialysis patients has a significant effect on the physical and psychological quality of life and mortality (7, 8). Insomnia is the predominant sleep disturbance in patients undergoing haemodialysis, with a prevalence ranging from 69% to 80% (9). Insomnia entails challenges in both initiating and sustaining sleep, premature awakening in the morning or during the night, and subpar sleep quality. It has also been reported that these individuals experience inconsistent sleep patterns, nightmares, morning headaches, and excessive daytime sleepiness (9).

Poor sleep is primarily influenced by the buildup of uremic toxins, anaemia, pain, and mental health challenges (10). It may also be attributed to restless leg syndrome and pruritus owing to uremia (11), sleep apnoea (12), and suboptimal diabetes management (9). Diabetes typically induces sleep disturbances, whereas sleep issues, such as inconsistent amounts of sleep, can exacerbate diabetes management, including diabetic retinopathy (9).

A series of studies conducted in different settings has identified several clinical conditions, demographic parameters, and laboratory indices of HD patients that contribute to sleep disturbances. Evidently, sleep disturbances are typically prevalent among patients with more comorbidities (6) and prolonged durations of haemodialysis (13), which heightens the incidence of symptoms and comorbidities, such as muscle cramps, peripheral neuropathy, and bone pain, resulting in worse sleep quality (14). Moreover, gender, age, educational attainment, and marital status have a significant relationship with the sleep quality of HD patients. Also, people who retired, had a greater number of dependents, and did not engage in exercise had exhibited significantly inferior sleep quality compared to other groups (6, 7, 15, 16). Further, higher risk factors for sleep disturbance include reduced levels of blood parathyroid hormone (PTH), 25-hydroxy vitamin D, albumin, and calcium (7, 16). Thus, numerous studies have clearly indicated that insomnia and suboptimal sleep quality are more common in HD patients and are aggravated by multiple risk factors across diverse demographics and settings. This work has confirmed that insufficient sleep is no longer a trivial health issue that can be overlooked.

Consequent effects of inadequate sleep are significantly correlated with exhaustion and diminished quality of life, often resulting in more serious, life-threatening conditions such as depression, weakened immunological function, and heightened risk of cardiovascular issues (17). Over 50% of patients undergoing haemodialysis have daytime somnolence and weariness, while over 25% exhibit symptoms of depression (18). Notably, patients experiencing sleep abnormalities are threefold more likely to suffer from depressive disorders compared to those without sleep issues (9). Furthermore, daytime sleepiness resulting from poor sleep quality is considered a crucial contributor to diminished focus and irritability (13), memory deterioration, and elevated anxiety levels (19) that result in a reduced quality of life (9). Remarkably, patients with reduced sleep duration, sleep disturbances, persistent feelings of unrest, moderate to regular use of sleeping medications, and sleep disorders are identified as having an elevated mortality risk (20).

However, sleep disturbances and overall poor sleep quality among patients still have not attracted the attention of the clinicians and related health professionals, leading to deficiencies in the effective diagnosis and management practices. Importantly, in low-resource settings like Sri Lanka, there is minimum attention to quality of life aspects of patients, which ultimately brings about ineffective treatment and mortality. A recent study has proposed that the prompt detection and diagnosis of sleep disorders in patients with end-stage kidney disease could enhance their survival prospects (21). Hence, it is vital to investigate and identify risk factors and novel predictors that contribute to poor sleep quality to facilitate early detection of sleep issues and recommend strategies and healthcare policies that can be established to improve the quality of life and survival of HD patients.

There is evidently a significant research gap on sleep quality in haemodialysis patients, especially in low-resource settings, with only a few extensive studies thoroughly investigating the determinants of inadequate sleep across various clinical and socio-demographic settings. Consequently, the present study sought to fill this research void and evaluate sleep quality among haemodialysis patients in a resource-limited context such as Sri Lanka. Additionally, the study examined new and potentially modifiable predictors of poor sleep quality, which specifically included demographic characteristics, dialysis-related factors, dietary factors, and biochemical parameters. The primary objective was to identify factors that can be monitored or targeted through clinical interventions to improve dialysis management, enhance patients’ quality of life, and ultimately contribute to better survival outcomes.

## 2 Materials and methods

### 2.1 Research design and setting

A descriptive cross-sectional design was employed to examine the overall status of sleep quality and predictors of poor sleep quality among hemodialysis patients in North Central Province. The study was conducted from January to December 2024 in dialysis centers of three government hospitals in Polonnaruwa, Anuradhapura, and Padaviya regions that provide free medical services to all citizens.

### 2.2 Sample

A random sample of 310 maintenance HD patients was selected for the study. Patients above the age of 18 years, who provided their consent to participate in the study and were able to converse with the investigators with no physical discomforts, were included in the study. Participants who were not willing to participate, suffered from critical conditions, and were cognitively unable to participate, were excluded.

### 2.3 Data collection tools

Data collection was conducted across four main categories using different tools and methods. Information for the questionnaires was recorded through the involvement of investigators, as interviewer-administered questionnaires.

1. Demographic and lifestyle information

Demographic information including age, level of education, income, employment status, distance from home to the hospital and time spent on travelling etc., and several important lifestyle habits like diet, appetite, daily water intake etc., were collected using a general questionnaire designed by the researchers.

2. Sleep quality

Participants’ sleep quality was assessed using the Pittsburgh Sleep Quality Index (PSQI). This tool contains seven components: subjective sleep quality, sleep latency, sleep duration, habitual sleep efficiency, sleep disturbances, use of sleep medication, and daytime dysfunction. There are 19 questions in this questionnaire, with a total score ranging from 0 to 21. According to the PSQI global score, patients who scored 5 or less considered to have normal sleep, and those who scored more than 5 were considered to have poor sleep. Hence, a better sleep quality is indicated by a lower score and the quality of sleep worsens with the increase in the score.

3. Dialysis-related information, calculations and medical history

Information like dialysis vintage, dialysis sessions per week, and vascular access, were recorded according to the responses of patients and also were confirmed with their dialysis records. Hemodialysis side effects and complications experienced by patients were also recorded. Urea Reduction Ratio (URR) and single pool Kt/V (spKt/V) were calculated to analyze the dialysis efficiency. Primary cause of CKD, year of diagnosis, and comorbidities of the patient, were obtained from the medical records.

4. Laboratory investigations

Several laboratory parameters including serum urea, creatinine, and electrolytes (Na, K) levels were analyzed both before and after dialysis. In addition, hemoglobin concentration, albumin, total protein, total calcium and phosphorus levels were analyzed from the blood samples collected before dialysis.

### 2.4 Ethical considerations

All the rules of ethics were observed, at every stage of the study. Ethical clearance for the study was obtained from the Ethics Review Committee, Faculty of Allied Health Sciences, University of Peradeniya, Sri Lanka (AHS/ERC/2023/044). Further, before initiating research procedures, written consent was obtained from all the participants after providing all information regarding the purpose of research, voluntary participation, and the possibility of withdrawing from the study at any stage. Their participation was voluntary, and throughout the study, the rules of confidentiality and anonymity were respected.

### 2.5 Statistical analysis

Descriptive statistics, including frequencies, percentages, means, and standard deviations, were performed to describe the study data. The normality of continuous variables was analysed using the Kolmogorov–Smirnov test. Inferential statistical methods were employed to study the relationships and differences among variables. Associations and correlations were determined using the chi-square test and Pearson correlation coefficient, respectively. Group mean differences were assessed using independent sample *t*-tests and one-way analysis of variance (ANOVA), as appropriate.

In this study, the main aim was to explore and explain the relationship between overall sleep quality and clinical, dialysis-related, and laboratory factors. Thus, multiple linear regression analysis was performed to maximize the ability to examine changes across the full range of sleep quality and identify potential predictors of poor sleep quality among CKD patients undergoing haemodialysis. A significance level of *p* < 0.05 was considered for all statistical tests. Data analysis was carried out using IBM SPSS version 25.0 (IBM Corp., Armonk, NY, USA) and Jamovi version 2.6.44 (R-based software).

## 3 Results

### 3.1 Descriptive analysis

#### 3.1.1 Demography and clinical details

A majority of 77.1% of the study sample was male individuals and the mean age of the population was 56 ± 11.1 years. Also, all participants of the study were receiving standard 4 h hemodialysis sessions on a regular basis. When considering the occupational status of the participants, 73.9% were unemployed or retired, and more than half of the subjects (59.3%) had only attended up to primary school. About half of the population were farmers and 37% of them had already abandoned farming due to their physical difficulties related to hemodialysis. As a result, more than half of the population (55.6%) has a very low monthly household income of less than 30,000 LKR (less than USD 97). According to the latest statistics, the average household income per month in Sri Lanka is 76,414 LKR (USD 247.27) (22), and 85.3% of the population had an income less than this amount.

A significant majority of the study subjects (79.1%) were spending more than 30 min or an hour in travelling to the dialysis center. The mean recovery time of the population, which is the time taken for patients to return to their usual physical and mental condition after dialysis was 4.9 ± 10.6 h, and 23.5% of participants presented physical discomforts that lasted for several hours, while for 12.6% individuals this lasted for 1 to 3 days.

The above findings on the descriptive analysis of the demographics, dialysis data and other medical details are illustrated in Table 1.

**Table 1:** Descriptive analysis of demographics, dialysis data and medical details.

Variable	Mean ±SD/ n (%)	Variable	Mean ±SD/ n (%)
Demographic and lifestyle information		Dialysis information and medical details	
1. Age (years)	56±11.1	8. Dialysis vintage (months)	30.3±31.5
≤ 40 years	28 (9.1)	< 1 year	98 (32.2)
41 – 60 years	167 (54.0)	1 - < 5 years	156 (51.3)
> 60 years	114 (36.9)	≥ 5 years	50 (16.4)
2. Gender		9. Dialysis Access	
Male	239 (77.1)	Permanent access	291 (96.4)
Female	71 (22.9)	Temporary access	11 (3.6)
3. Marital status		10. Dialysis sessions per week	
Single	25 (8.2)	< Twice a week	107 (36.5)
Married	278 (90.8)	Twice a week	169 (57.7)
Other	03 (1.0)	Thrice a week	17 (5.8)
4. Level of education		11. Primary cause of CKD	
Primary	175 (59.3)	CKDu	195 (64.6)
Secondary	113 (38.3)	Diabetic Nephropathy	64 (21.2)
Higher education	7 (2.4)	Other	43 (14.2)
5. Occupational status		12. Cardiovascular diseases	
Unemployed/retired	224 (73.9)	Present	250 (82.5)
Employed	79 (26.1)	Absent	53 (17.5)
6. Time spent on travelling to the dialysis center (Minutes)	66.2±43.5	13. No. of comorbidities	1.33±0.91
<30 min	58 (20.9)	0	48 (15.8)
30 – 60 min	113 (40.8)	1 - 2	223(73.6)
> 60 min	106 (38.3)	≥ 3	32 (10.6)
7. Appetite on dialysis days		14. Recovery time (hours)	4.9±10.6
Same as other days	78 (25.9)	15. Dialysis adequacy	
Low	171 (56.8)	Kt/V	1.5±0.5
High	52 (17.3)	Urea Reduction Ratio (%)	68.6±11.9

#### 3.1.2 Sleep quality

Table 2 represents the scores of the PSQI tool used to assess the sleep quality of the population. The reliability of the questionnaire was measured with the Cronbach alpha index which showed a high reliability (Cronbach alpha = 0.844).

**Table 2:** Pittsburgh Sleep Quality Index (PSQI) scores of the study population.

Components	Mean score ± SD	Grading of the score – *n* (%)			
		0 No problem	1 Moderate problem	2 Severe problem	3 Very severe problem
1. Subjective sleep quality	1.4 ± 0.9	39 (13)	127 (42.4)	94 (31.4)	39 (13)
2. Sleep latency	2 ± 1.1	37 (12.4)	67 (22.4)	51 (17.1)	144 (48.2)
3. Sleep duration	1.8 ± 1.2	58 (19.4)	61 (20.4)	64 (21.4)	116 (38.8)
4. Habitual sleep efficiency	1.8 ± 1.1	49 (16.4)	76 (25.4)	55 (18.4)	119 (39.8)
5. Sleep disturbances	1.2 ± 0.5	15 (5)	200 (66.9)	83 (27.8)	1 (0.3)
6. Use of sleeping medication	0.2 ± 0.7	268 (89.6)	7 (2.3)	12 (4)	12 (4)
7. Daytime dysfunction	1.3 ± 0.7	31 (10.4)	157 (52.5)	93 (31.1)	18 (6)

Final score	Mean ± SD	Good sleep quality (≤ 5)	Poor sleep quality (>5)
Global PSQI Score	9.86 ± 4.2	50 (16.7)	249 (83.3)

The mean global PSQI score of the study population was 9.86 ± 4.2, where a notable majority of 83.3% had poor sleep quality. The mean habitual sleep efficiency of the population was 65.01 ± 18.98, and 39.8% of the participants had a habitual sleep efficiency of <65%. Further, a majority of 38.8% sleep only for <5 h, where some individuals get just a sleep of 1–2 h. The patients are so much troubled with inadequate sleep to the extent that it has become a mental burden. The first complaint of most patients, when inquired about their daily troubles and discomforts due to dialysis, was that they were unable to get a good night sleep.

**Figure 1:**
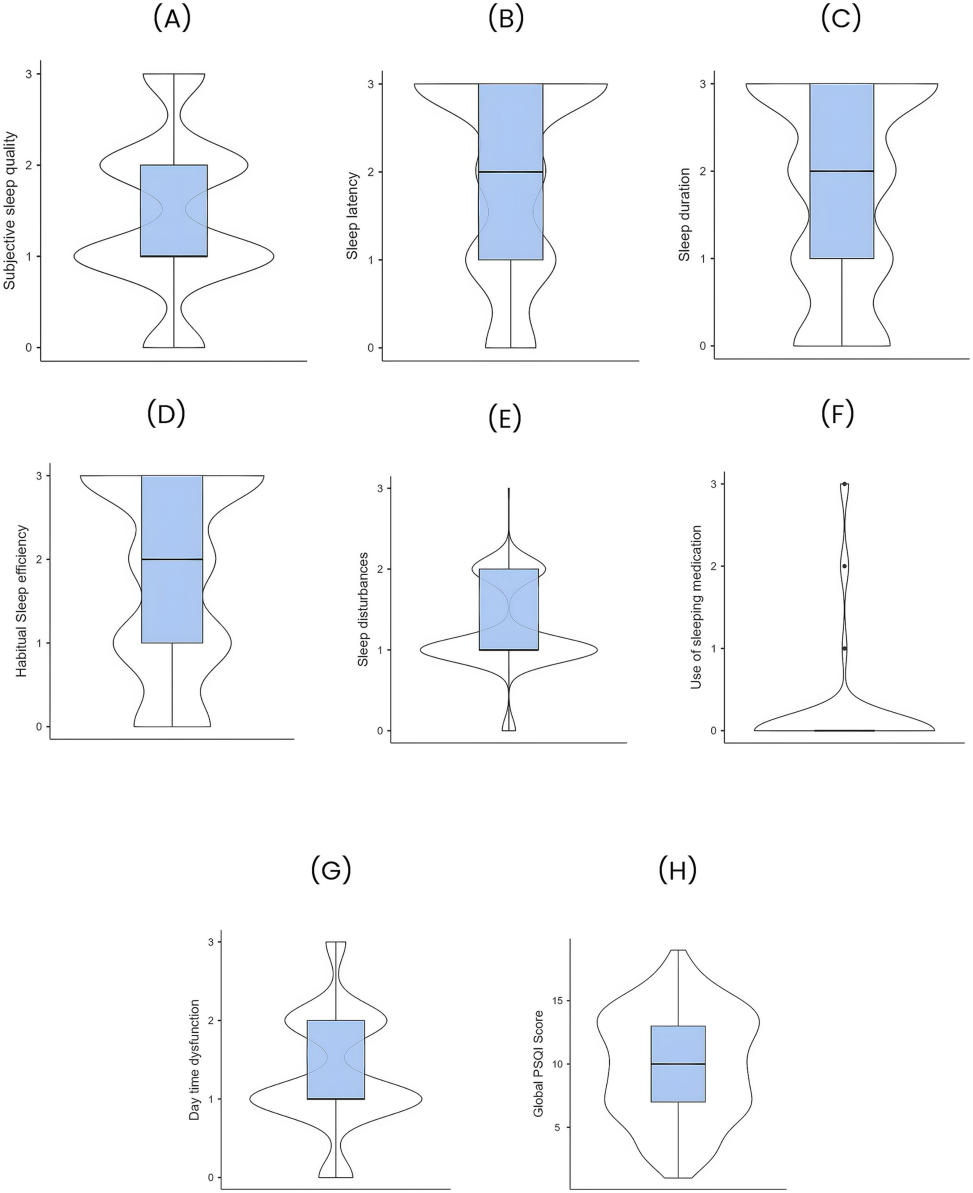
Distribution of Pittsburgh sleep quality index (PSQI) scores of the study population. **(A)** Subjective sleep quality **(B)** Sleep latency, **(C)** Sleep duration, **(D)** Habitual sleep efficiency, **(E)** Sleep disturbances, **(F)** Use of sleep medication, **(G)** Daytime dysfunction, and **(H)** Global PSQI score.

Figure 1 is a graphical representation of the distribution of PSQI scores of the population.

### 3.2 Chi-square analysis

Dialysis center (*p* < 0.01) and dialysis sessions per week (p < 0.01) depicted significant associations with the sleep quality of patients. Specifically, patients on twice weekly dialysis suffered from worse sleep quality comparatively to other groups. Further, presence of several hemodialysis side effects (23) such as muscle cramps (*p* = 0.02), muscle pain (*p* = 0.003), hypertension (*p* = 0.002), shortness of breath (*p* = 0.001), fluid overload (*p* = 0.026), joint pain/joint disease (*p* = 0.036), faintness/dizziness (*p* = 0.019), numbness in hands or feet (*p* = 0.01), nausea (*p* = 0.046) and headache (*p* = 0.007), were strongly associated with poor sleep quality among participants. High post dialysis serum urea level (*p* = 0.011), high serum phosphorus levels (*p* = 0.016) and low hemoglobin levels (*p* = 0.046) presented significant associations with diminished sleep quality. In addition, dialysis vintage (*p* = 0.054) and appetite (*p* = 0.055) of patients had associations of marginal significance with the sleep quality, where patients with comparatively low appetite on dialysis days and individuals who have been engaged in dialysis for 1 to 5 years, were presented with poor sleep quality.

### 3.3 Correlation analysis

Correlation analysis was conducted using Pearson correlation coefficient after analyzing the normality of the variables using Kolmogorov–Smirnov test.

The global PSQI score depicted a significant positive correlation with the number of dialysis side effects and complications of patients (*r* = 0.351, *p* < 0.01), recovery time (*r* = 0.255, *p* < 0.01) and post dialysis urea concentration (*r* = 0.129, *p* = 0.027). Further, overall sleep quality of patients showed a significant negative correlation with the post dialysis sodium concentration (*r* = −0.16, *p* = 0.006), hemoglobin concentration (*r* = −0.16, *p* = 0.005), urea reduction ratio (*r* = −0.13, *p* = 0.023) and Kt/V (*r* = −0.21, *p* = 0.001).

### 3.4 Analysis of independent sample *t* test

Normality check for the variables were assessed using the Kolmogorov–Smirnov test and the independent sample t test was selected to compare mean values of the variables.

The differences of mean global PSQI score among the different categories of variables are presented in Table 3. Accordingly, the type of hemodialysis access shows a marginal significance with regard to differences of sleep quality in the population. Specifically, patients with temporary hemodialysis access were recognized to have poor sleep quality. Moreover, patients who had high post dialysis creatinine levels were also having sleeping difficulties.

**Table 3:** Results of the independent sample t test

Variable	Mean Global PSQI Score ± SD	p	Variable	Mean Global PSQI Score ± SD	p
1. Hemodialysis access		0.054	10. Emotional distress		< 0.01
Permanent access	9.78±4.25		Absent	9.24 ±4.17	
Temporary access	12.4 ±2.68		Present	10.95± 4.06	
2. Muscle cramps		0.002	11. Faintness/dizziness		< 0.01
Absent	8.75±4.2		Absent	8.91 ±4.1	
Present	10.35±4.12		Present	10.88± 4.09	
3. Muscle Pain		<0.01	12. Numbness in hands/feet		< 0.01
Absent	8.68± 4.19		Absent	9.03 ±4.12	
Present	10.53±4.07		Present	10.78 ±4.12	
4. Itching/Dry skin		0.006	13. Nausea		< 0.01
Absent	9.16± 4.17		Absent	9.31± 4.15	
Present	10.49± 4.15		Present	11.23± 4.01	
5. Hypertension		0.034	14. Headaches		< 0.01
Absent	9.27±4.57		Absent	8.98± 4.12	
Present	10.29± 3.86		Present	10.87± 4.06	
6. Shortness of Breath		< 0.01	15. Cramps		< 0.01
Absent	8.68± 4.03		Absent	8.76 ±4.07	
Present	11.57 ±3.86		Present	10.74± 4.1	
7. Washed out/drained		< 0.01	16. Joint diseases/joint pain		< 0.01
Absent	9.38 ±4.08		Absent	9.07± 4.16	
Present	11.30 ±4.27		Present	10.69± 4.1	
8. Fluid overload		0.005	17. Hemoglobin concentration		0.001
Absent	1.38 ±4.18		Low	10.25±4.18	
Present	10.85± 4.09		Normal	8.27±3.97	
9. Chest pain		0.004	18. Post HD Creatinine level		0.009
Absent	9.46± 4.1		Normal	6.75 ±3.25	
Present	11.2 ±4.31		High	9.95± 4.18	

### 3.5 One-way analysis of variance

One way ANOVA test was conducted after performing the normality check for the variables.

**Figure 2:**
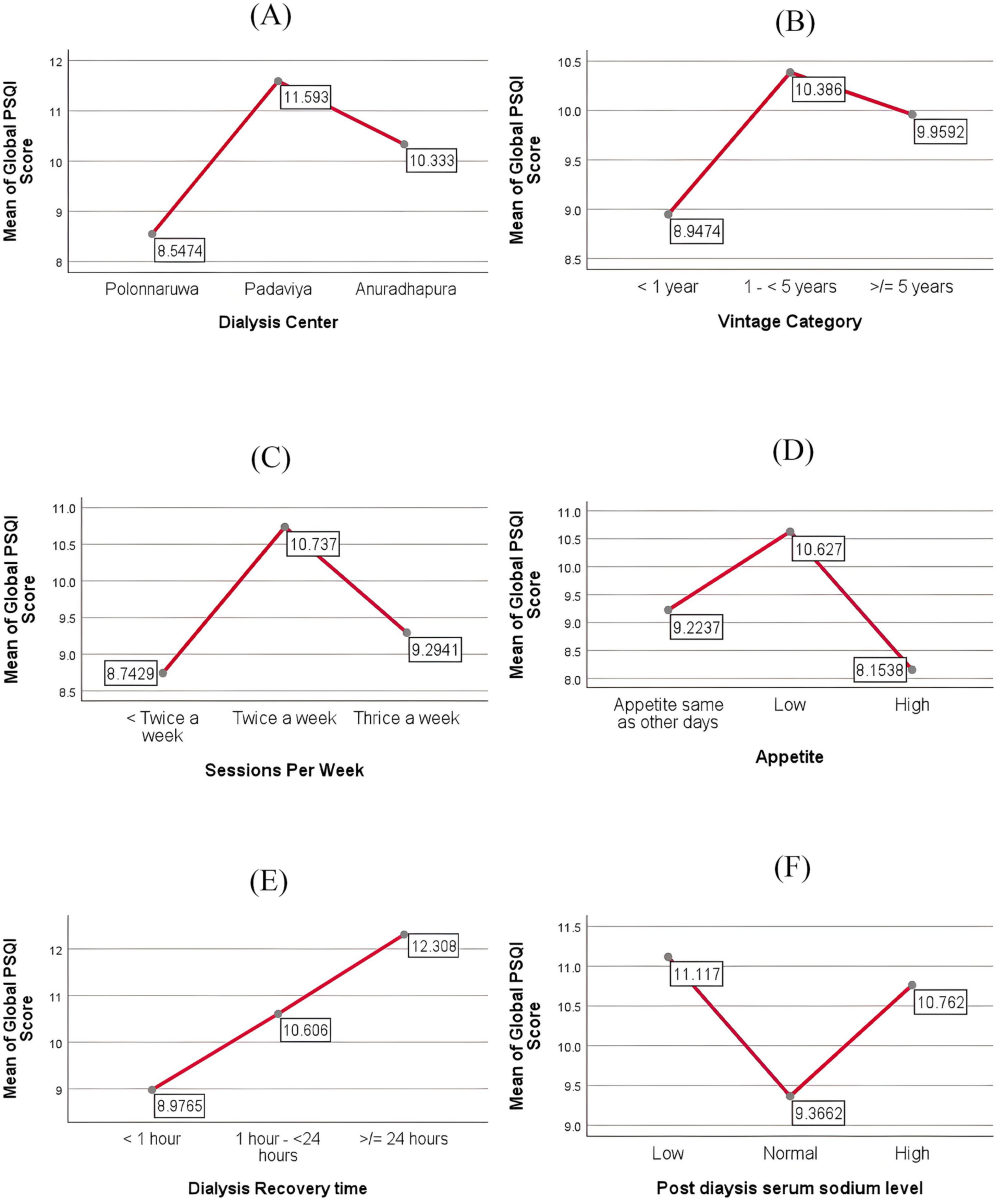
Mean plots of categorical variables **(A–F)**. Dialysis center **(A)**, dialysis vintage category **(B)**, dialysis sessions per week **(C)**, appetite of patients on the dialysis days **(D)**, recovery time **(E)**, post dialysis sodium level **(F)**.

The mean plots of several variables that depicted significant differences in the mean Global PSQI score among their categories are presented in Figure 2. Mean differences among the categories of the variables and their values of significance are presented in Table 4.

**Table 4:** Findings of one way ANOVA analysis.

Variable	Mean difference (reference category – other categories)	Significance (p)
1. Dialysis Center		
Polonnaruwa^a^		
Padaviya**	–3.045**	< 0.01
Anuradhapura**	–1.786**	0.005
2. Dialysis Vintage category		
< 1 year^a^		
1 year to < 5 years*	–1.438*	0.024
≥ 5 years	–1.012	
3. Sessions per week		
< Twice a week^a^		
Twice a week**	–1.994**	< 0.01
Thrice a week	–0.551	
4. Appetite		
Low^a^		
Same as other days*	1.404*	0.037
High**	2.473**	0.001
5. Recovery time		
< 1 hour^a^		
1 hour to < 24 hours*	–1.629*	0.013
≥ 24 hours**	–3.331**	< 0.01
6. Post HD Na level		
Low^a^		
Normal*	1.750*	0.011
High	0.355	

^a^ – Represent the reference category.

*/** - Represent the categories that depict a significant difference from the mean PSQI of the reference category.

*p < 0.05, **p < 0.01.

According to the analysis, a noteworthy fact is that patients with a recovery time of more than 1 h have significantly high mean values of Global PSQI score, and it is even higher when it comes to more than 24 h, compared to those with a recovery time less than 1 h, indicating a significant increase of sleep problems with the increase in recovery time.

### 3.6 Analysis of multiple linear regression

After performing the normality check using the Shapiro–Wilk test, multiple linear regression was performed to identify significant predictors of poor sleep quality among the population. Collinearity was assessed using the Variance Inflation Factor (VIF), and standard model diagnostics were performed to ensure the selected model provided the best fit for the data.

According to the model depicted in Table 5 (*R*^2^ = 0.386, *N* = 240), hospital/dialysis unit (*B* = 1.96, *p* = 0.043), dialysis access (*B* = 3.89, *p* = 0.018), total number of side effects and complications of a patient (*B* = 0.31, *p* < 0.001), appetite (*B* = 1.94, *p* = 0.001) and post dialysis serum urea concentration (*B* = 0.05, *p* = 0.012) were identified as significant predictors of poor sleep quality.

**Table 5:** Output of Multiple Linear Regression that presents the predictors of poor sleep quality in the study population

Model Coefficients – Global PSQI Score						
Predictor	Estimate	SE	p	Stand. Estimate	95% Confidence Interval	
					Lower	Upper
Interceptᵃ	11.4580	8.6482	0.187			
Age	0.0194	0.0229	0.399	0.0496	–0.06603	0.16524
Gender:						
Female – Male	–1.0422	0.6425	0.106	–0.2482	–0.54971	0.05337
Hospital:						
Padaviya – Polonnaruwa*	1.9629	0.9654	**0.043**	0.4674	0.01434	0.92049
Anuradhapura - Polonnaruwa	–0.7916	1.0868	0.467	–0.1885	–0.69859	0.32158
Primary cause of CKD:						
Diabetic Nephropathy – CKDu	0.7000	0.6413	0.276	0.1667	–0.13429	0.46764
Other – CKDu	0.3697	0.7095	0.603	0.0880	–0.24496	0.42102
Dialysis vintage:						
1 - < 5 years – < 1 year	0.3438	0.5710	0.548	0.0819	–0.18613	0.34987
≥5 years – < 1 year	–0.5173	0.7811	0.509	–0.1232	–0.48976	0.24341
HD sessions per week:						
Twice a week – < Twice a week	0.9391	0.9087	0.303	0.2236	–0.20284	0.65008
Thrice a week – < Twice a week	–0.9979	1.2072	0.409	–0.2376	–0.80419	0.32894
Dialysis Access:						
Temporary Access – Permanent Access*	3.8930	1.6381	**0.018**	0.9270	0.15822	1.69581
Total number of side effects and complications**	0.3126	0.0544	**<.001**	0.3467	0.22788	0.46561
Recovery time (hours)	0.0430	0.0235	0.068	0.1093	-0.00831	0.22690
Appetite:						
Low – Appetite same as other days**	1.9389	0.5875	**0.001**	0.4617	0.18599	0.73742
High – Appetite same as other days	–0.7698	0.7424	0.301	–0.1833	–0.53173	0.16511
BMI:						
Normal – Underweight	–0.6379	0.6068	0.294	–0.1519	–0.43669	0.13287
Overweight – Underweight	–0.2425	0.8932	0.786	–0.0578	–0.47696	0.36145
Obese I – Underweight	–0.9753	0.8579	0.257	–0.2322	–0.63487	0.17040
Obese II – Underweight	1.6046	1.4983	0.285	0.3821	–0.32110	1.08530
Post HD Urea level (mg/dl)*	0.0524	0.0208	**0.012**	0.1771	0.03872	0.31557
Post HD Creatinine level (mg/dl)	–0.3360	0.1743	0.055	–0.1540	–0.31144	0.00346
Post HD Sodium level (mmol/l)	–0.0521	0.0594	0.381	–0.0580	–0.18839	0.07234

ᵃ Represents reference level.

*p < 0.05, **p < 0.01.

Significant *p* values (*p* < 0.05 and *p* < 0.01) are indicated in bold.

## 4 Discussion

Sleep is a fundamental component of quality of life (QOL), significantly influencing physical, mental, and emotional well-being. Dialysis patients face specific health challenges that heighten the importance of healthy sleep. Inadequate sleep can aggravate underlying medical illnesses and impede recovery, whereas sufficient sleep can enhance general quality of life and treatment efficacy. However, numerous studies indicate that poor sleep quality is a significant complaint among dialysis patients, with an incidence ranging from 40% to 85% (17). This underscores the imperative for healthcare professionals to prioritise the sleep quality and associated psychological concerns of haemodialysis patients. Further, it is critical to conduct in-depth research to identify and understand the underlying factors that lead to diminished sleep quality for the purpose of implementing management strategies.

This study is the first to analyse the incidence and determinants of sleep quality among hemopodialysis patients in Sri Lanka. The research was conducted in CKDu endemic areas of Sri Lanka, involving 310 haemodialysis patients from dialysis centres in the Anuradhapura and Polonnaruwa districts. Further, it contributes to the existing literature on the subject by identifying significant novel predictors that determine poor sleep quality of haemodialysis patients and also by emphasising the sleep issues and overall sleep quality of HD patients residing in resource-constrained environments.

The current study sample exhibited a notably high prevalence of poor sleep quality of 83.3% (*n* = 249). This prevalence is considerably greater than the findings of recent research conducted in various countries (6, 24, 25), which can be attributed to the healthcare policies constrained by limited resource settings in the country. However, as per the findings of similar studies, poor sleep latency is the most prominent dimension of PSQI in the population (26, 27), suggesting that the majority of the population takes longer to fall asleep. As a result, the sleep duration of the majority of patients is far below the recommended range, and some patients only get a sleep of 1–2 h due to the added problems of waking frequently throughout the night and waking up too early in the morning. Such behaviour makes the patients devoid of necessary physical and mental rest and diminishes their physical and psychological well-being, ultimately leading to ineffective treatments.

Notably, poor fluid management and fluid overload were identified as major adversities in the population. According to the concerns of patients, frequent disturbances to their sleep at night are tempting them to drink more water, aggravating fluid imbalances and fluid overload. Consequently, subpar fluid management practices among the patients have led to many adverse health outcomes in the population that diminish their quality of life and even clinical prognosis.

Being in a low-income country, patients face several issues pertaining to inadequate haemodialysis, lack of minimal resources, financial problems when affording medicine and transport, improper transport methods, etc. Thus, inadequate sleep aggravates the problems for these patients, resulting in feelings of dissatisfaction, discomfort, sorrow, powerlessness, and diminished independence and concern regarding their health status, which can ultimately lead to depression and anxiety.

There are numerous potential causes for sleep disruptions, including the renal disease and the dialysis procedure. The current study analysed the underlying factors contributing to the notably high prevalence of poor sleep quality in the study population, identifying several dialysis-related factors, dietary factors, and some biochemical results as prominent.

According to the 2015 clinical practice guidelines of the National Kidney Foundation, the conventional form of dialysis is thrice-weekly in-center haemodialysis (28). Nonetheless, due to economic limitations and an increasing demand from a burgeoning patient population, a low-income country such as Sri Lanka is incapable of offering thrice-weekly dialysis at the restricted government dialysis centers. Consequently, thrice-weekly dialysis is infrequently administered to specific important patients, whereas the majority of patients receive twice-weekly haemodialysis. The heightened demand and restricted resource availability in specific locations of the country have compelled some patients to attend sessions solely once a week. The study encompassed patients from a government hospital in Polonnaruwa, where most patients participate in dialysis sessions every 4 to 5 days (fewer than twice weekly), and from two hospitals in Padaviya and Anuradhapura, where the majority of patients receive dialysis biweekly. The data indicate that individuals undergoing twice-weekly haemodialysis experience markedly inferior sleep quality compared to those receiving less frequent haemodialysis. This outcome may be associated with psychological factors arising from individuals’ personal challenges, including the disruption of their typical social life and routine due to their medical condition; the necessity to abandon their occupation entirely to adhere to the dialysis schedule, which has intensified the financial strain related to transportation and medication costs; and the presence of depression and anxiety. Nevertheless, the impact of psychological issues, such as depression and anxiety, on patients’ sleep quality and general quality of life is not thoroughly examined in the present study. Consequently, research is already underway to evaluate this issue concerning HD patients in resource-limited environments. Furthermore, the impact of low income and financial strain on patients’ quality of life, particularly regarding sleep quality, necessitates comprehensive examination and should be considered in forthcoming research.

The designated tertiary hospital in the Polonnaruwa district, established specifically for the treatment of chronic kidney disease (CKD) patients, exhibits a lower incidence of sleep disturbances and overall sleep quality issues. In contrast, the hospitals in Anuradhapura and Padaviya, which lack specialised facilities and dedicated personnel for CKD patients, report a significantly higher prevalence of sleep quality problems. The notable disparity in sleep quality among patients at these three dialysis centers may arise from the differing physical and human resources accessible to them.

Moreover, individuals undergoing dialysis for 1 to 5 years have more sleep disturbances compared to those with shorter or longer dialysis durations. While certain studies have reported a deterioration in sleep quality associated with prolonged dialysis vintage (29), the present study revealed a rather divergent situation. Patients in the initial phases of haemodialysis experience a degree of relief from dialysis treatments, as they attain physical comfort following diminished fluid overload and relatively fewer limitations on water consumption and eating habits. However, as patients undergo treatments for extended durations of 1 to 3 years, they begin to encounter difficulties in adhering to the dialysis schedule and managing the expenses that come with it. Additionally, they begin to worry over their constrained lifestyle and the inconveniences of the dialysis method and excessively contemplate their health status and future prospects. Such thoughts may directly affect their physical and psychological health, thereby influencing their sleep quality and general quality of life. After successfully completing four to five years of haemodialysis, patients gradually adjust to the dialysis schedule and integrate it into their regular routine. Patients who have had dialysis for 5 years or more exhibit a relatively positive mentality and fewer concerns regarding their illness.

Numerous studies have highlighted a significant inverse relationship between dialysis adequacy and the sleep quality of haemodialysis patients (30, 31). The present study cohort also reported a similar result. Nonetheless, the dialysis adequacy of patients is not frequently assessed in numerous healthcare settings across the country due to various flaws in the healthcare system. In light of the study’s findings and the significance of dialysis adequacy in influencing sleep quality, which affects the overall quality of life of haemodialysis patients, it is essential to discover and implement solutions for continuous evaluation of patients’ dialysis adequacy.

Furthermore, no comprehensive study has analysed the relationship between vascular access and sleep quality in HD patients. However, the current analysis identified vascular access to dialysis as a major predictor of poor sleep quality; patients who engaged in dialysis via temporary catheters experienced comparatively low sleep quality. In the prevailing medical setting of the country, patients with planned haemodialysis also have to commence haemodialysis with temporary catheters and sometimes have to use them for longer periods, like 6 months to 1 year, due to insufficient resources, a shortage in the workforce, and long waiting lists for the AVF (arteriovenous fistula) surgery. It was observed that patients face both physical and mental difficulties when they undergo dialysis treatments with temporary catheters, which also pose risks of infection. Thus, in light of the above findings, it is important to focus on resource allocations and health policy amendments to alleviate the negative effects of temporary vascular access on the quality of life of HD patients in Sri Lanka.

It is widely acknowledged that haemodialysis patients experience significant symptom burdens that adversely affect their quality of life and mortality (5). The current study assessed the prevalence of various haemodialysis side effects and complications within the study population (23). It was revealed that the majority of these side effects, particularly muscle cramps, muscle pain, hypertension, shortness of breath, fluid overload, joint diseases/joint pain, faintness/dizziness, numbness in the hands or feet, emotional distress, nausea, and headache, were significantly correlated with individuals’ sleep quality. The total number of side effects and complications in patients was determined to be a significant predictor of their sleep quality. The research conducted by (6) also identified symptom distress in patients as a significant factor affecting overall sleep quality.

Furthermore, a significant variable influenced by the patients’ symptom burden is the dialysis recovery time (DRT). It is referred to as the period of time required for patients to return to their typical physical and psychological condition following a session of haemodialysis, which is a crucial indicator that reflects patients’ QOL (32). The findings of the current study revealed a significant correlation between the recovery time and sleep quality of the patients. Importantly, every component of the PSQI (Pittsburgh Sleep Quality Index), especially sleep latency, sleep duration, and sleep disturbances, grew worse with the increase in the population’s recovery time. No comprehensive investigation has been undertaken to evaluate the relationship between the dialysis recovery time and sleep quality of haemodialysis patients. However, the current research findings point out that prolonged recovery time exacerbates sleep disturbances and diminishes overall sleep quality, highlighting the necessity for further research.

The current research demonstrated a notable association between sleep quality and appetite in individuals undergoing haemodialysis, aligning with several studies that indicated an important relationship between dietary components and the sleep quality of dialysis patients (27, 33). Participants with a diminished appetite on dialysis days reported significantly lower sleep quality compared to those with a high appetite or a normal regular appetite.

Further, the outcomes of several laboratory investigations were recognised as important predictors of sleep quality among the study sample. Increased post-dialysis serum urea levels, diminished sodium levels, and reduced haemoglobin concentrations demonstrated a significant correlation with the sleep quality of patients. Elevated urea levels signify uremia, characterised by the accumulation of uremic toxins that induce weariness, physical discomfort, mood fluctuations, confusion, and other psychological disturbances in patients, ultimately compromising their sleep quality (31, 34). The impact of low sodium levels on sleep quality illustrates the importance of electrolyte balance in sustaining optimal physical and mental health in patients (35). Low haemoglobin levels are recognised as a primary factor contributing to poor sleep in the current study, supported by findings from multiple other research studies (36–38). Moreover, elevated phosphorus levels adversely affected the sleep quality of the population. The correlation between phosphorus levels and sleep quality is intricate and has to be particularly examined. However, the high phosphorus level can affect vascular and bone health, creating an indirect effect on the sleep quality of patients (27).

The nation’s prevailing health practices lack a systematic plan for conducting laboratory examinations for patients, owing to resource limitations and financial constraints. The study’s findings underscore the necessity of continuous monitoring of patients’ biochemical profiles to enhance sleep quality, which significantly affects the overall physical and psychological well-being of haemodialysis patients.

It is essential to recognise the limits of the current study in order to propose thorough research that mitigates these issues. Despite utilising a multicenter research methodology, the majority of the study group comprised patients from economically disadvantaged families and resource-limited environments in the country. Therefore, future studies should involve participants from various economic, geographical, and social backgrounds to enhance outcomes through comparative analysis across these groups. Furthermore, owing to constrained resources, the study was unable to examine the influence of certain biochemical measures (such as vitamins and inflammatory markers) and particular clinical aspects (special questionnaires regarding comorbidities and weariness) on sleep quality. Consequently, the authors advocate for future research incorporating additional characteristics that may serve as potential causal factors. Furthermore, the study failed to include specific instruments to evaluate the effects of pruritus, restless leg syndrome, and particular medical issues such as depression and anxiety, as its primary aim was to investigate the predictors of reduced sleep quality, primarily associated with demographic factors, dialysis variables, dietary habits, and biochemical indicators. Investigations are underway to examine the correlation between patients’ anxiety and depression levels and the sleep quality and general quality of life of HD patients. Moreover, longitudinal research designs may be utilised to examine the fluctuations in the sleep quality of patients over time, influenced by factors such as dialysis vintage, dietary modifications, fluid balance, and dialysis adequacy.

## 5 Conclusion

Given the high prevalence of poor sleep quality among haemodialysis patients and its substantial impact on their overall quality of life, it is crucial for researchers and healthcare providers to address this often-overlooked issue in clinical practice. The findings of the current study reveal a significant burden of sleep disturbances, which contribute to both physical and psychological complications, meaning that they require immediate attention and targeted management. Multiple factors, including dialysis-related variables, dietary habits, appetite changes, and various biochemical parameters, have been identified as significant determinants of poor sleep quality and require careful monitoring and follow-up to improve patient outcomes. Healthcare providers can mitigate sleep problems by optimising dialysis treatment parameters, maintaining proper fluid balance, managing comorbid conditions, promoting sleep hygiene practices, and implementing pharmacological interventions when necessary. Importantly, treatment strategies should be individualised based on patients’ specific needs and the underlying causes of their sleep disturbances. Additional research, encompassing clinical intervention studies, is essential to augment comprehension and formulate effective strategies, ultimately enhancing treatment efficacy, prognosis, quality of life, and life expectancy in this susceptible population.

## Data Availability

The raw data supporting the conclusions of this article will be made available by the authors, without undue reservation.
